# A 66-Year-Old Woman with a Progressive, Longitudinally Extensive, Tract Specific, Myelopathy

**DOI:** 10.1155/2016/4125294

**Published:** 2016-11-21

**Authors:** Elizabeth O'Keefe, Katherine E. Schwetye, John Nazarian, Richard Perrin, Robert E. Schmidt, Robert Bucelli

**Affiliations:** ^1^Department of Neurology, Division of Physical Medicine and Rehabilitation, Washington University School of Medicine, Campus Box 8518, 4444 Forest Park Blvd., St. Louis, MO 63108, USA; ^2^Department of Pathology & Immunology, Division of Neuropathology, Saint Louis University School of Medicine, 1402 South Grand Blvd., Saint Louis, MO 63104, USA; ^3^Department of Radiology, Wake Radiology, 3949 Browning Place, Raleigh, NC 2760, USA; ^4^Department of Pathology & Immunology, Division of Neuropathology, Washington University School of Medicine, Campus Box 8118, 660 S. Euclid Ave., St. Louis, MO 63110, USA; ^5^Department of Neurology, Divisions of Neuromuscular and General Neurology, Washington University School of Medicine, Campus Box 8111, 660 S. Euclid Ave., St. Louis, MO 63110, USA

## Abstract

A 66-year-old woman presented with progressive lancinating pain and sensory deficits attributable to a myelopathy of unclear etiology. Spinal cord magnetic resonance imaging showed a longitudinally extensive T2-hyperintense lesion of the dorsal columns. Comprehensive serum, urine, and cerebrospinal fluid analyses failed to identify an etiology. Empiric intravenous methylprednisolone and intravenous immunoglobulin were of no benefit and serial screens for an occult malignancy were negative. She developed dysesthesias and allodynia affecting her entire body and lost the use of her arms and legs due to severe sensory ataxia that was steadily progressive from onset. She opted against additional aggressive medical management of her condition and passed away on hospice eleven months after symptom onset. Autopsy revealed findings most consistent with polyphasic spinal cord ischemia affecting the dorsal and lateral white matter tracts and, to a lesser extent, adjacent gray matter. The underlying etiology for the progressive vasculopathy remains unknown. Spinal cord ischemia affecting the posterior spinal cord is rare and to our knowledge this case represents the only instance of a progressive spinal cord tractopathy attributable to chronic spinal cord ischemia.

## 1. Introduction

Dorsal column myelopathies commonly present with a chief complaint of ataxia and incoordination. Exam findings include loss of proprioception (leading to sensory ataxia), vibration sense, two-point discrimination and light touch with preserved pain, and temperature sensation [1, 2]. The differential diagnosis includes infectious, inflammatory (including autoimmune, paraneoplastic, and demyelinating), metabolic, toxic, and vascular (e.g., dural arteriovenous fistula) etiologies which may be seen as longitudinally extensive T2 hyperintensity localized to the dorsal columns on magnetic resonance imaging (MRI, see* “Differential Diagnosis of Chronic Myelopathy”*) [2–11]. Although exceedingly rare, posterior cord syndromes can also be associated with an ischemic infarct of the spinal cord [5]. However, cases of dorsal spinal cord ischemia reported in the literature are associated with typical temporal features of a vascular disorder, including acute onset and monophasic course [5, 6].

*Differential Diagnosis of Chronic Myelopathy*

*Inflammatory/Autoimmune*

- Vasculitis
- Neuro-Behcet's
- Sjögren's
- Neurosarcoidosis
- Lupus
- Paraneoplastic (most common antibodies)
  - amphiphysin immunoglobulin G [IgG]
  - collapsin response-mediator protein 5 IgG
  - Purkinje-cell cytoplasmic autoantibody type 1, 2
  - antineuronal nuclear autoantibody (ANNA-1), and ANNA-3
- Neuromyelitis optica (NMO) and NMO spectrum disorders
  - Aquaporin-4 IgG
  - Myelin-oligodendrocyte glycoprotein (MOG) IgG (rare in adults)
  - Seronegative NMO (controversial entity)
- Multiple sclerosis
- Sarcoidosis
- CLIPPERS (chronic lymphocytic inflammation with pontine perivascular enhancement responsive to steroids)
- Acute disseminated encephalomyelitis (ADEM) (rare in adults)

*Vascular*

- Spinal cord infarction
- Arteriovenous malformations
- Diffuse atherosclerosis
- Dural arteriovenous fistula

*Hereditary/Degenerative*

- Primary lateral sclerosis
- Hereditary spastic paraparesis
- Spinocerebellar ataxias
- Mitochondrial disorders
- Adult polyglucosan body disease

*Compressive/Structural*

- Tumor
- Atlantoaxial subluxation
- Syrinx

*Metabolic*

- B12 deficiency
- Copper deficiency
- Vitamin E deficiency

*Toxic*

- Radiation myelopathy
- Nitrous oxide

*Infectious*

- Human immunodeficiency virus (HIV)
- Human T-lymphotropic virus (HTLV)-1
- Neurosyphilis
- Schistosomiasis
- Brucellosis

*Differential Diagnosis of a Dorsal Column Myelopathy*

*Metabolic*

- B12 deficiency
- Copper deficiency

*Vascular*

- Posterior spinal artery infarction
- Vascular malformations
- AVFs

*Infectious*

- Neurosyphilis
- HIV

See [1–6, 26–28].

We present the case of a woman with a predominantly progressive posterior cord syndrome of unclear etiology. Autopsy findings suggest that chronic, progressive microvasculopathy should be considered in the differential diagnosis of a dorsal column myelopathy when alternative, more common, etiologies have been ruled out.

## 2. Case Presentation

A 66-year-old woman presented with lancinating pain, allodynia, and numbness that steadily ascended from her feet to the level of her umbilicus over a three-month period. There was no clinical history to suggest an episodic or relapsing-remitting course. Her past medical history was significant for tobacco use, hypertension, poorly controlled type II diabetes mellitus, chronic kidney disease, chronic obstructive pulmonary disease, breast cancer (ductal carcinoma, otherwise unspecified; 13 years after mastectomy and reconstruction, not requiring chemotherapy), and a ruptured hepatic abscess (8 years after abdominal wall reconstruction). She had no known history of nitrous oxide exposure. Her neurologic examination showed normal mental status and cranial nerves and a normal motor exam when utilizing visual feedback to guide movement of the muscle groups being tested. She had no visual field deficits on bedside testing and there were no other clinical signs or symptoms of optic neuropathy. Joint position sense was severely impaired, with a corresponding severe sensory ataxia and impaired upper and lower extremity vibration sense (distal worse than proximal). Reflexes were symmetrically brisk (3+). Bilateral Babinski and Hoffman's signs were elicited.

**Figure 1:**
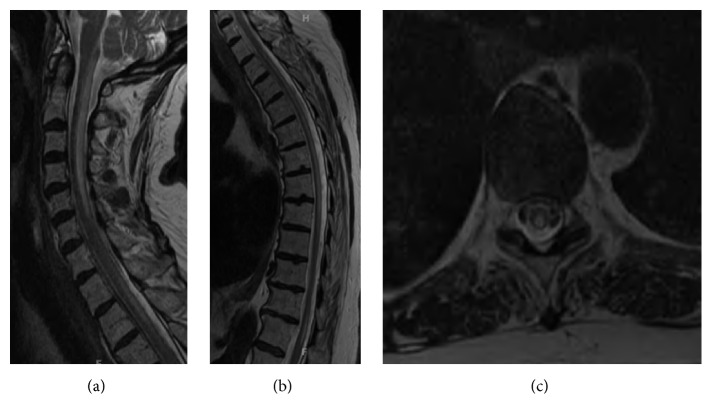
Sagittal T2-weighted MR images through the cervical (a) and thoracic (b) spine demonstrate diffuse, hyperintense signal in the posterior columns, extending nearly the entire length of the spinal cord. An axial T2W image at T9-T10 (c) demonstrates the cross-sectional extent of the signal abnormality.

MRI of the cervical and thoracic spine was obtained at the time of presentation and demonstrated mild, diffuse atrophy of the cord and a diffuse hyperintense signal on T2-weighted images within the dorsal columns, extending from the craniocervical junction to T12 (Figure 1). A low creatinine clearance precluded a contrast enhanced MRI of the spine and spinal angiography to evaluate for a dural arteriovenous fistula (AVF). MRI of the brain showed punctate T2 white matter hyperintensities, a finding felt to be most consistent with small vessel disease in this clinical context.

## 3. Clinical Discussion

The differential diagnosis for a dorsal column predominant “tractopathy” is broad and includes infectious, inflammatory/autoimmune/demyelinating, metabolic, toxic, and vascular (e.g., dural arteriovenous fistula or rarely ischemic infarction) etiologies (see* “Differential Diagnosis of Chronic Myelopathy”*). A lesion involving the dorsal columns symmetrically and extending over so many spinal levels with commensurate cord atrophy might suggest a metabolic process such as subacute combined degeneration from vitamin B12 deficiency or copper-deficiency myeloneuropathy. However, chronic sequelae from syphilis (tabes dorsalis) or HIV (vacuolar myelopathy) could have the same appearance. In contrast, homogenous extension over such a long spinal segment, without contrast enhancement, would be unusual for a demyelinating disease, such as multiple sclerosis or neuromyelitis optica (NMO), or for a neoplasm. Although intravascular B cell lymphoma (IVBCL) can present with longitudinally extensive spinal cord lesions, this neoplasm would be expected to cause expansion of the cord and is rarely predominantly tract specific [13].

Paraneoplastic myelopathies, on the other hand, are commonly tract specific [8, 14–16]. Antineuronal nuclear antigen 1 (ANNA-1, also known as anti-Hu), antineuronal nuclear antigen 2 (ANNA-2), antineuronal antigen 3 (ANNA-3), antiamphiphysin, and anticollapsing response mediator protein 5 (CRMP-5) are among the most common antibodies identified in patients with paraneoplastic myelopathies [17]. However, antibodies directed against a number of other surface and intracellular antigens have also been reported [8, 18]. Paraneoplastic myelopathies have been reported in association with many forms of cancer, the most common being breast and lung cancer [17].

## 4. Diagnostic Evaluation and Management

An extensive evaluation for metabolic/toxic (vitamin B12 deficiency, copper deficiency, vitamin E deficiency, and heavy metals), infectious (bacterial (syphilis), viral (HIV, fungal, and tuberculosis)), and autoimmune etiologies (NMO, MS, and sarcoidosis) was nondiagnostic (Table 1). Neurosyphilis was excluded by the absence of pleocytosis on CSF analysis and a negative serum RPR and CSF VRDL. A purified-protein derivative skin test for tuberculosis was negative. A serum paraneoplastic autoantibody panel including antistriated muscle, antiacetylcholine receptor (ganglionic neuronal), voltage-gated calcium channel P/Q type, anti-CRMP-5 IgG, antineuronal nuclear 1, 2, 3, anti-Purkinje cell cytoplasmic types 1 and 2 and TR, antiamphiphysin, antiglial nuclear, antineuronal voltage-gated potassium channel, and anti-GAD65 antibodies, as well as serum aquaporin-4 IgG was performed by Mayo Clinic, Rochester, Minnesota [12]. This panel was borderline positive for N-type voltage-gated calcium channel (VGCC) antibodies at a titer of 0.03 nM on an initial sample and 0.04 nM on a repeat sample sent two months after her initial presentation. The patient underwent extensive screening for an occult malignancy, including a whole body positron emission tomography/computed tomography (PET/CT) scan, mammography, and colonoscopy, all of which were negative/nondiagnostic. PET scan did show FDG-avid lesions in lung parenchyma and paratracheal nodes which prompted biopsy of the paratracheal nodes which were negative for malignancy and showed no granulomatous disease. Electromyography and nerve conduction studies showed a mild, axonal, length-dependent sensorimotor polyneuropathy. A quadriceps biopsy performed to screen for IVBCL showed chronic neurogenic changes of increased fiber size variation, pyknotic nuclear clumps, and grouped atrophy with fiber type grouping. A skin biopsy also showed no evidence of intravascular B cell lymphoma. Empiric intravenous methylprednisolone (3 of 5 grams completed over 3 days, with the final two grams held due to significant hyperglycemia) and intravenous immunoglobulin (IVIg, 2 g/kg) were of no benefit. After discharge her disease continued to steadily progress and she was readmitted 3 weeks later with worsened upper limb sensory ataxia, rendering her unable to feed herself or live independently. Her allodynia and pain had similarly escalated and were no longer responsive to symptomatic therapies. Two additional IVIg (1 g/kg/month) treatments were of no benefit. Given her intractable pain, bedbound status with a hospitalization for pneumonia, and 100% dependency for all activities of daily living, she was placed on comfort care through home hospice. She passed away eleven months after onset of symptoms.

## 5. Neuropathologic Findings

**Figure 2:**
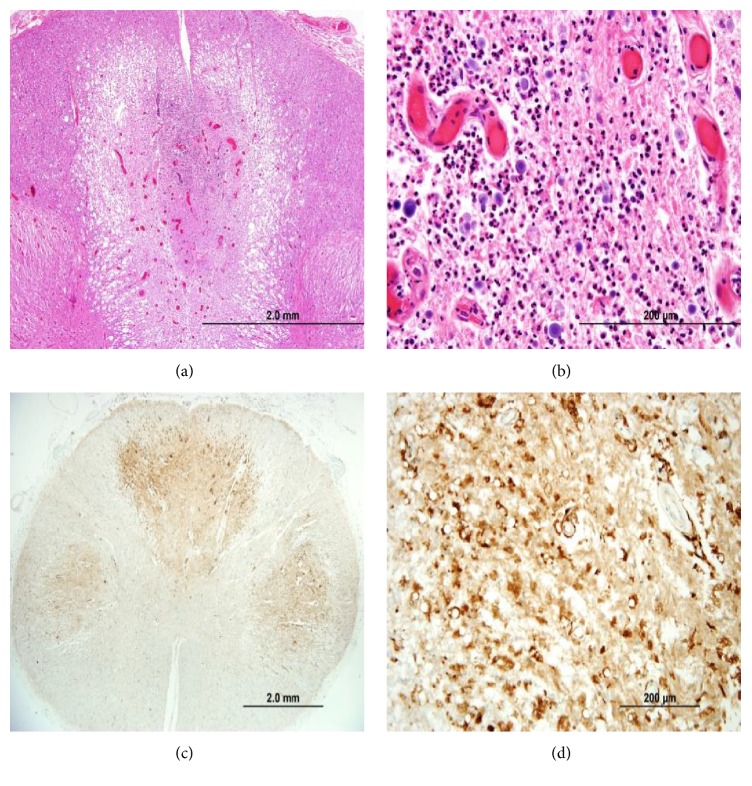
Acute and subacute ischemic changes in the spinal cord. (a, b) Intermediate-power and high-power photomicrographs of dorsal columns in a section with neutrophilic infiltrates and reactive vessels (original magnifications, ×40 and ×400, resp.; H-E stain); (c) low-power photomicrograph of spinal cord stained with antibody against CD68 to demonstrate foamy macrophages and microglia, primarily within dorsal columns and lateral white matter tracts (original magnification, ×20; CD68 immunostain); (d) higher-power photomicrograph of the dorsal columns showing CD68-positive macrophages and microglia (original magnification, ×200; CD68 immunostain).

**Figure 3:**
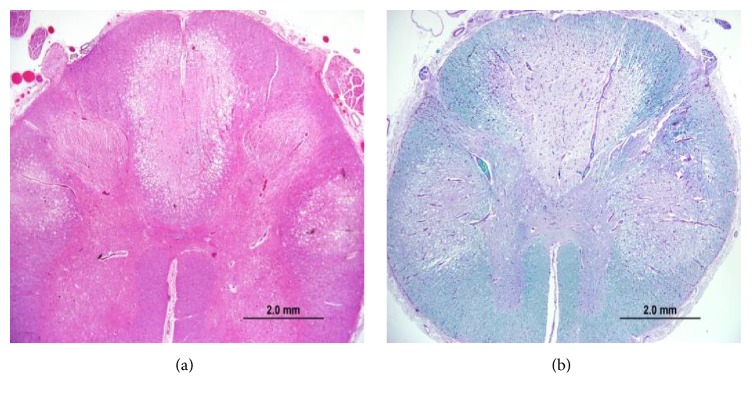
Histologic sections of spinal cord demonstrate extensive white matter pathology. (a) Low-power photomicrograph of spinal cord with dorsal column and lateral white matter degeneration (original magnification, ×20; hematoxylin-eosin [H-E] stain); (b) low-power photomicrograph of spinal cord stained with Luxol fast blue-periodic acid Schiff (LFB-PAS) histochemistry to demonstrate loss of myelinated axons (original magnification, ×20; LFB-PAS stain).

**Figure 4:**
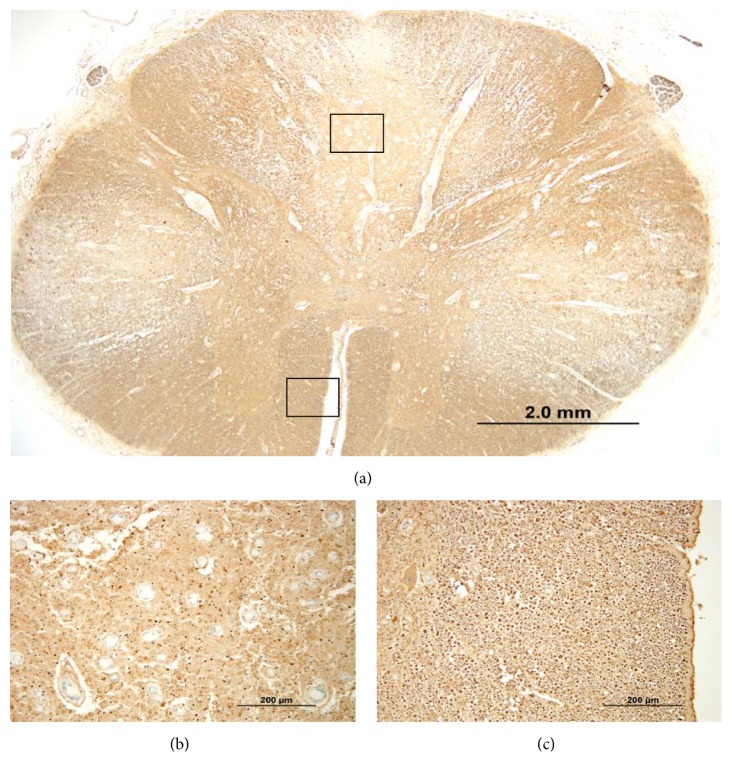
Spinal cord stained immunohistochemically with antibody to neurofilament (NF) demonstrates regional axonal loss; (a) low-power photomicrograph demonstrates areas of axonal loss most pronounced in the dorsal (upper boxed area) and lateral white matter tracts, in contrast to ventral spinal cord (lower boxed area) (original magnification, ×20; NF immunostain); (b, c) higher-power photomicrographs of boxed areas to demonstrate axonal loss in dorsal columns ((b) upper boxed area from (a)) with relative axonal preservation in ventral area ((c) lower boxed area from (a)) (original magnifications, ×200; NF immunostain).

**Figure 5:**
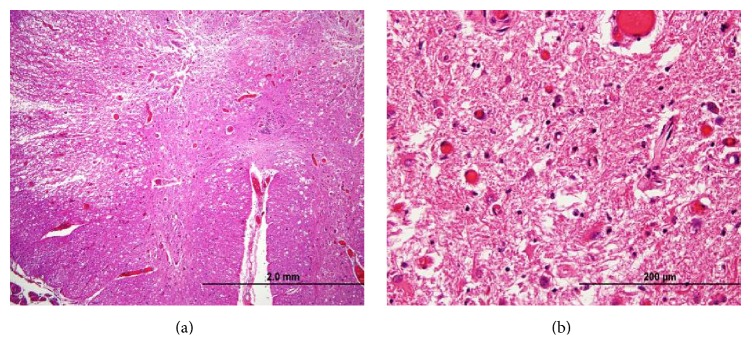
Gray matter damage. (a) Lower-power photomicrograph of gray matter adjacent to ventrolateral tracts (original magnification, ×40; H-E stain); (b) higher-power photomicrograph of adjacent gray matter with neuronal loss and gliosis (original magnification, ×400; H-E stain).

Postmortem examination confirmed the cause of death as bilateral pneumonia with multiple flora and foreign material (consistent with aspiration) and found severe diffuse atherosclerosis within the aorta from the arch to the bifurcation, with evidence of ulceration. No neoplasm was detected. Gross examination of the spinal cord revealed atrophy and mild discoloration. Microscopic examination of the spinal cord showed a complex polyphasic myelopathy affecting dorsal and lateral white matter tracts and, to a lesser extent, adjacent gray matter. More specifically, the dorsal columns showed degeneration in a medial-to-lateral gradient, with evidence of acute, subacute, and chronic changes. The more acute/subacute changes included a neutrophilic infiltrate limited to the medial portions of the dorsal columns, appearing in association with acute/subacute necrosis, reactive/reparative vasculature, activated microglia, and numerous foamy macrophages (Figure 2). The chronic changes included loss of myelinated axons (Figures 3 and 4). Similar chronic changes were observed within the lateral corticospinal tracts, with extension into the ventrolateral white matter and adjacent gray matter (Figure 5). The superficial white matter and ventral-most areas of white and gray matter were relatively spared.

Outside the areas of injury, blood vessels in the spinal cord showed only moderate arteriolosclerosis, without inflammation or thrombosis. Similar arteriolosclerosis was evident in the brain and meninges. The brainstem showed only very focal coarse vacuolation of the right spinothalamic tract and ventral spinocerebellar tract in the lateral medulla; other ascending and descending tracts were unremarkable. Sections of sampled dorsal root ganglia showed no evidence of nodules of Nageotte or of ganglion cell damage or loss. The feeding vessels of the vertebral and spinal arteries, which originate from the aorta, were not collected during prosection, so subsequent histopathologic examination of these vessels was not possible.

This pattern of spinal cord injury, involving the dorsal columns in a gradient of severity from medial (worst) to lateral (less severe) and also involving the dorsolateral white matter, correlated with the clinical history of ascending sensory symptoms. Coupled with damage to gray matter, this pattern also raised suspicion for chronic ischemia/infarction as an etiology for the patient's myelopathy.

## 6. Discussion

This case is unique and instructive for both the spatial (predominantly posterior columns on imaging with involvement of additional tracts on tissue analysis) and temporal (subacute-to-chronic and steadily progressive) pattern of myelopathy, presumed secondary to a polyphasic ischemic process. Typically, spinal cord infarction presents with an acute onset of deficits that vary based on the territories involved. Overall, nontraumatic spinal cord ischemia or infarction accounts for only ~1% of total strokes [17]. Usually, spinal cord ischemia/infarction results from hypoperfusion of the thoracolumbar spinal cord, which can occur in the settings of atherosclerotic disease of the aorta or vertebral arteries, dissecting aortic aneurysm, aortic surgical intervention, spinal trauma, or systemic hypotension [5]. Less common etiologies include vasculitis, cocaine abuse, presumed fibrocartilaginous embolism, decompression sickness, subclavian vein catheterization, sympathectomy, thoracoplasty, celiac plexus neurolysis, lumbar epidural anesthesia, intrathecal injection of lidocaine, dural arteriovenous fistula, intramedullary arteriovenous malformation, single radicular artery ligation, vertebral angiography, and renal artery embolization [5, 19]. Infarction of the posterior columns can be related to vertebral artery dissection and trauma of the spine or surgery. The histopathologic sequelae of ischemia are variable and depend on both the level and extent of vascular compromise. Autopsy findings in patients affected by spinal cord infarction often include preferential involvement of gray matter; however, in a large series, the most severe cases also showed white matter necrosis [18, 20]. Other reports describe involvement of both gray and white matter [21]. With respect to vascular territories, infarction localized predominantly to the region supplied by the posterior spinal arteries is very rare and the pathophysiology has not been clearly defined [17, 21]. Because the posterior columns have a consistent blood supply from two posterior spinal arteries and the large number of anastomoses, it is hypothesized that this region is often spared [5, 6, 22]. However, in cases of hypoperfusion, a watershed effect may take precedence [17]. In the spinal cord, there is a dorsal watershed zone characterized by a capillary network where the penetrating branches of the anterior spinal artery meet the penetrating branches of the posterior spinal arteries and branches of the circumferential pial network [17]. This vascular anatomy may also contribute to the neurologic manifestations of chronic spinal cord ischemia in the setting of arteriovenous malformations and AVFs.

Signs and symptoms of spinal cord ischemia may vary in intensity and evolve over a few days before deficits become fixed. This patient presented with a subacute-to-chronic history of pain and severe sensory ataxia, and MRI findings supported localization to the posterior cord; accordingly, our initial differential diagnosis focused on common causes of this form of myelopathy. This patient did not fit the typical case of spinal cord infarction as she had no acute inciting event and it is uncommon for spinal cord infarction to preferentially involve the dorsal columns [5, 6, 17, 22, 23]. Furthermore, these acute inciting events have a much stronger association with anterior, rather than posterior, spinal cord infarctions in the literature [17]. A spinal angiogram (to evaluate for a dural AVF) was not possible in the setting of the patient's low creatinine clearance.

The underlying etiology of this patient's apparent polyphasic spinal cord infarction remains unclear. The lack of an inflammatory CSF profile, nodular contrast enhancing lesions in the spine (typical of sarcoidosis), nondiagnostic paratracheal lymph node biopsy guided by results of a full body PET scan, and lack of pathologic features of noncaseating granulomas on complete autopsy, all argued against neurosarcoidosis. The patient's serum anti-aquaporin-4 IgG was negative and other than radiologic evidence of a longitudinally extensive lesion of the dorsal columns there were no clinical features suggestive of NMO or NMOSD. Radiological and postmortem examinations identified severe atherosclerosis in the aorta and visceral blood vessels, but there was no definitive evidence of thrombosis or vasculitis to explain the more acute changes. Given the symmetric, tract specific changes on imaging, symmetric clinical deficits, and the progressive course, a paraneoplastic myelopathy remains possible, despite the lack of an identified occult malignancy at autopsy or on PET/CT scan. Although VGCC autoantibodies have been reported in cases of paraneoplastic myelopathy they are not specific to this disorder; and, given the low titers, this finding has unclear clinical significance [14, 16]. Nonetheless, given the many factors suggestive of a paraneoplastic etiology and the possibility of an unidentified antibody-mediated disorder, the patient underwent extensive screening for an occult malignancy, including a whole body positron emission tomography/computed tomography (PET/CT) scan, mammography, and colonoscopy. PET/CT scans certainly improve the likelihood of identifying an occult malignancy in suspected paraneoplastic disorders but are not 100% sensitive and in this case serial PET scans did not identify a malignancy [24, 25]. The unusual findings of ischemia/infarction suggest that if this were paraneoplastic in nature, it would be associated with a novel form of a paraneoplastic microvasculopathy [14]. The pathologic features of paraneoplastic myelopathies are not well characterized, precluding a comparison of our findings to others reported in the literature.

## 7. Conclusion

Chronic, progressive microvasculopathy should be considered in the differential diagnosis of a progressive myelopathy with imaging evidence of a dorsal column tractopathy when alternative, more common, etiologies have been ruled out. Given the strong suspicion for an occult malignancy in our case and the strong clinical resemblance to previously reported cases of paraneoplastic dorsal column myelopathy, we propose that an immune mediated microvasculopathy be considered in the list of potential pathophysiologic mechanisms for paraneoplastic myelopathies. To our knowledge, no comparable cases exist in the literature and additional cases are necessary to further test this hypothesis, emphasizing the importance of postmortem examinations in this unfortunate patient population.

## Figures and Tables

**Table 1:** Relevant data.

*Plasma/serum/urine*		
Vitamin B12 (211–911 pg/mL)	524 pg/mL	
Methylmalonic acid (≤0.40 nmol/L)	0.31 nmol/L	
Homocysteine (5–15 *µ*mol/L)	11.7 *µ*mol/L	
Vitamin E (5.5–17.0 mg/L)	17.7 mg/L	
TSH (0.35–5.50 mcIU/mL)	1.07 mcIUnits/mL	
Free T4 (0.9–1.8 ng/dL)	1.12 ng/dL	
*β*2-Microglobulin (1.1–2.5 mg/L)	4.4 mg/L	
ANA, ANCA, anti-dsDNA, ENA	Negative	
24-hour urine heavy metals (mercury, lead, arsenic)	Negative	
24-hour urine zinc (300–600 mcg/24 h)	909 mcg/24 hr	
24-hour urine copper (15–60 mcg/24 h)	22 mcg/24 hr	
Serum paraneoplastic panel × 2^*∗*^	0.03 and 0.04 nmol/L	
Negative except for calcium channel ab N-type (<0.03)		
*Pathology*		
CSF cytology	Negative	
Fine needle aspiration of right paratracheal lymph node	Negative	
Skin biopsy	Negative for lymphoma	
Quadriceps biopsy	Negative for lymphoma	
*Microbiology*		
HIV	Negative	
HTLV-1	Negative	
RPR	Negative	
Mycobacteria (AFB) culture and acid fast stain	Negative	
PPD	Negative	
Blood cultures	Negative	
Urine cultures	Negative	
Respiratory cultures from bronchoalveolar lavage	Negative	
CSF culture × 2	Negative	
Fungal culture of blood	Negative	
*CSF*	*Jun 14*	*Aug 14*
Glucose	113 mg/dL	120 mg/dL
Protein (15–45 mg/dl)	30 mg/dL	41 mg/dL
Nucleated cells (cells/*µ*L)	0	0
Red blood cells	0	0
CSF IgG index (≤0.85)	0.6	0.62
CSF specific oligoclonal bands	0	0
CSF paraneoplastic panel^*∗*^	Negative	
CSF cytology		
*Electromyography (EMG)/ nerve conduction study*		
Length-dependent sensorimotor axonal polyneuropathy with small sural sensory nerve action potential amplitudes and EMG evidence of subacute and chronic neurogenic changes limited to a distal leg muscle.		

TSH, thyroid stimulating hormone; HIV, human immunodeficiency virus; HTLV, human T-lymphotropic virus; RPR, rapid plasma reagin; ANA, anti-nuclear antibody; ANCA, anti-neutrophil cytoplasmic antibody; dsDNA, double-stranded DNA; ENA, extractable nuclear antigen.

^*∗*^Paraneoplastic panel: antistriated muscle; antiacetylcholine receptor (ganglionic neuronal); anti-voltage-gated calcium channel binding P/Q type; CRMP-5 IgG; antineuronal nuclear 1, 2, 3; Purkinje cell cytoplasmic types 1 and 2 and TR; amphiphysin, antiglial nuclear; antineuronal voltage-gated potassium channel; and anti-GAD65 antibodies. CSF panel tested with indirect immunofluorescence assay and serum panel tested with indirect immunofluorescence assay and radioimmunoassay (RIA) [12].

## References

[1] Lindsay K. W. I. B. R. C. J. V. G. (1991). *Neurology and Neurosurgery Illustrated*.

[2] Jacob A., Weinshenker B. G. (2008). An approach to the diagnosis of acute transverse myelitis. *Seminars in Neurology*.

[3] Kumar N. (2012). Metabolic and toxic myelopathies. *Seminars in Neurology*.

[4] Petito C. K., Navia B. A., Cho E.-S., Jordan B. D., George D. C., Price R. W. (1985). Vacuolar myelopathy pathologically resembling subacute combined degeneration in patients with the acquired immunodeficiency syndrome. *The New England Journal of Medicine*.

[5] Weidauer S., Nichtweiß M., Hattingen E., Berkefeld J. (2015). Spinal cord ischemia: aetiology, clinical syndromes and imaging features. *Neuroradiology*.

[6] Novy J., Carruzzo A., Maeder P., Bogousslavsky J. (2006). Spinal cord ischemia: clinical and imaging patterns, pathogenesis, and outcomes in 27 patients. *Archives of Neurology*.

[7] Berger J. R., Sabet A. (2002). Infectious myelopathies. *Seminars in Neurology*.

[8] Flanagan E. P., Keegan B. M. (2013). Paraneoplastic myelopathy. *Neurologic Clinics*.

[9] Wingerchuk D. M., Lennon V. A., Lucchinetti C. F., Pittock S. J., Weinshenker B. G. (2007). The spectrum of neuromyelitis optica. *The Lancet Neurology*.

[10] Andersen O. (2000). Myelitis. *Current Opinion in Neurology*.

[11] Caragine L. P., Halbach V. V., Ng P. P., Dowd C. F. (2002). Vascular myelopathies-vascular malformations of the spinal cord: presentation and endovascular surgical management. *Seminars in Neurology*.

[12] Mayo Medical Laboratories (2016). *Paraneoplastic Autoantibody Evaluation, Serum*.

[13] Hidekazu Y., Keisuke I., Masashi H. (2015). A case of acute progressive myelopathy due to intravascular large B cell lymphoma diagnosed with only random skin biopsy. *Clinical Neurology*.

[14] Flanagan E. P., McKeon A., Lennon V. A. (2011). Paraneoplastic isolated myelopathy: clinical course and neuroimaging clues. *Neurology*.

[15] Jiao Y., Zhang W., Li X. (2014). Longitudinally extensive spinal cord lesion: etiology and imaging features. *Zhonghua Yi Xue Za Zhi*.

[16] Flanagan E. P., Lennon V. A., Pittock S. J. (2011). Autoimmune myelopathies. *Continuum (Minneap Minn)*.

[17] Ropper A. H., Samuels M. A., Klein J. P., Ropper A. H., Samuels M. A., Klein J. P. (2014). Diseases of the spinal cord. *Adams & Victor's Principle's of Neurology*.

[18] Ishizawa K., Komori T., Shimada T. (2005). Hemodynamic infarction of the spinal cord: involvement of the gray matter plus the border-zone between the central and peripheral arteries. *Spinal Cord*.

[19] Rubin M. N., Rabinstein A. A. (2013). Vascular diseases of the spinal cord. *Neurologic Clinics*.

[20] Duggal N., Lach B. (2002). Selective vulnerability of the lumbosacral spinal cord after cardiac arrest and hypotension. *Stroke*.

[21] Wolf H. K., Anthony D. C., Fuller G. N. (1990). Arterial border zone necrosis of the spinal cord. *Clinical Neuropathology*.

[22] Shamji M. F., Maziak D. E., Shamji F. M., Ginsberg R. J., Pon R. (2003). Circulation of the spinal cord: an important consideration for thoracic surgeons. *Annals of Thoracic Surgery*.

[23] Cheshire W. P., Santos C. C., Massey E. W., Howard J. F. (1996). Spinal cord infarction: etiology and outcome. *Neurology*.

[24] McKeon A., Apiwattanakul M., Lachance D. H. (2010). Positron emission tomography-computed tomography in paraneoplastic neurologic disorders: systematic analysis and review. *Archives of Neurology*.

[25] Patel R. R., Subramaniam R. M., Mandrekar J. N., Hammack J. E., Lowe V. J., Jett J. R. (2008). Occult malignancy in patients with suspected paraneoplastic neurologic syndromes: value of positron emission tomography in diagnosis. *Mayo Clinic Proceedings*.

[26] Pandit L. (2015). Neuromyelitis optica spectrum disorders: an update. *Annals of Indian Academy of Neurology*.

[27] Wingerchuk D. M., Banwell B., Bennett J. L. (2015). International consensus diagnostic criteria for neuromyelitis optica spectrum disorders. *Neurology*.

[28] Tobin W. O., Weinshenker B. G., Lucchinetti C. F. (2014). Longitudinally extensive transverse myelitis. *Current Opinion in Neurology*.

